# Differences in MEF2 and NFAT Transcriptional Pathways According to Human Heart Failure Aetiology

**DOI:** 10.1371/journal.pone.0030915

**Published:** 2012-02-17

**Authors:** Raquel Cortés, Miguel Rivera, Esther Roselló-Lletí, Luis Martínez-Dolz, Luis Almenar, Inmaculada Azorín, Francisca Lago, José Ramón González-Juanatey, Manuel Portolés

**Affiliations:** 1 Cardiocirculatory Unit, Research Center, Hospital Universitario La Fe, Valencia, Spain; 2 Cardiology Unit, Hospital Universitario La Fe, Valencia, Spain; 3 Experimental Neurology, Research Center, Hospital Universitario La Fe, Valencia, Spain; 4 Molecular and Cellular Cardiology Unit, Institute of Biomedical Research, and Department of Cardiology, Hospital Clínico Universitario, Santiago de Compostela, Spain; 5 Cell Biology and Pathology Unit, Research Center, Hospital Universitario La Fe, Valencia, Spain; Cardiovascular Research Institute Maastricht, Maastricht University, Netherlands

## Abstract

**Background:**

Ca^2+^ handling machinery modulates the activation of cardiac transcription pathways involved in heart failure (HF). The present study investigated the effect of HF aetiology on Ca^+2^ handling proteins and NFAT1, MEF2C and GATA4 (transcription factors) in the same cardiac tissue.

**Methodology and Principal Findings:**

A total of 83 hearts from ischemic (ICM, n = 43) and dilated (DCM, n = 31) patients undergoing heart transplantation and controls (CNT, n = 9) were analyzed by western blotting. Subcellular distribution was analyzed by fluorescence and electron microscopy. When we compared Ca^+2^ handling proteins according to HF aetiology, ICM showed higher levels of calmodulin (24%, p<0.01), calcineurin (26%, p<0.01) and Ca^2+^/Calmodulin-dependent kinase II (CaMKIIδ_b_ nuclear isoform 62%, p<0.001) than the CNT group. However, these proteins in DCM did not significantly increase. Furthermore, ICM showed a significant elevation in MEF2C (33%, p<0.01), and GATA4 (49%, p<0.05); also NFAT1 (66%, p<0.001) was increased, producing the resultant translocation of this transcriptional factor into the nuclei. These results were supported by fluorescence and electron microscopy analysis. Whereas, DCM only had a significant increase in GATA4 (52%, p<0.05). Correlations between NFAT1 and MEF2C in both groups (ICM r = 0.38 and DCM r = 0.59, p<0.05 and p<0.01, respectively) were found; only ICM showed a correlation between GATA4 and NFAT1 (r = 0.37, p<0.05).

**Conclusions/Significance:**

This study shows an increase of Ca^2+^ handling machinery synthesis and their cardiac transcription pathways in HF, being more markedly increased in ICM. Furthermore, there is a significant association between MEF2, NFAT1 and GATA4. These proteins could be therapeutic targets to improve myocardial function.

## Introduction

Heart failure (HF) is caused by diverse conditions which reduce the efficiency of the myocardium through overloading or damage. Over time, these stimuli will produce changes to the heart itself, such as enlargement of ventricles and hypertrophy (ventricular remodeling) [1], [2], activating a molecular response in cardiomyocytes that involves an enhanced protein synthesis, up-regulation of fetal cardiac genes, and induction of immediate-early genes [3].

Numerous studies have implicated intracellular calcium (Ca^2+^) as a critical mediator in the regulation of left ventricular remodeling in HF [4], [5]. Changes in intracellular Ca^2+^ ion concentrations regulate the activity of several related proteins, kinases and phosphatases, among them the ubiquitous Ca^2+^-binding proteins, calmodulin (CaM), the Ca^2+^/Calmodulin-dependent kinase II (CaMKII), and calcineurin (CaN), a Ca^2+^/Calmodulin-dependent phosphatase.

Elevated intracellular Ca^2+^ and the resulting Ca^2+^/CaM complex will activate CaMKII and CaN, which play an important role in cardiac function (mediate cardiac hypertrophy response to myocyte stretch or increased loads). Both enzymes respond to dysregulated calcium signaling, as an increase in their expression and activity in failing human myocardium and in animal models with cardiac hypertrophy and HF [6]–[8]. Many major pathways for pathological remodeling converge on a set of transcriptional regulators, such as nuclear myocyte enhancer factor 2 (MEF2), nuclear factor of activated T cells (NFAT) and GATA binding protein 4 (GATA4) [9]–[11]. Furthermore, histone deacetylases (HDAC) play a critical role in the modulation of hypertrophic growth by inhibiting the activity of MEF2 [12].

There are different activation pathways in the expression of these transcriptional factors: (1) MEF2 transcriptional activity is repress by HDAC4s and becomes active in presence of CaMKII which promotes the export of HDAC from the nucleus [13], [14]; and (2) the activation of NAFT, a hyperphosphorylated cytosolic protein, is regulated through control of its subcellular localization. An elevation in intracellular Ca^2+^ increases the activity of CaN, which dephosphorylates the NFAT molecule and allows its import into the nucleus [15]. In addition, the NFAT interacts with the cardiac-restricted zinc finger transcription factor GATA4, resulting in synergic activation of cardiac transcription [9].

Previous data show the relevance of increased levels of both Ca^2+^/calmodulin-dependent enzymes, and these transcriptional factors, in the development of a hypertrophic phenotype [6], [13], [15]. However, to date most of these studies have been performed *in vitro* or in animal models [7], [13], [16] and the simultaneous analysis of the different activation pathways has not been performed yet. Therefore, the present study investigates the levels of CaM, CaN and CaMKIIδ, predominant isoform in the heart [17], in dilated (DCM) and ischemic cardiomyopathy (ICM) human left ventricular myocardium. Furthermore, we determine the potential relationships between these proteins on the transcriptional factors, NFAT, MEF2 and GATA4, in the same cardiac human tissue.

## Materials and Methods

### Collection of samples

Experiments were performed with left ventricular samples from 43 patients with ischemic cardiomyopathy (ICM) and 31 with dilated cardiomyopathy (DCM) undergoing cardiac transplantation. Clinical history, hemodynamic studies, ECG, Doppler echocardiography, and coronary angiography data were available on all these patients. All patients were functionally classified according to the New York Heart Association criteria (NYHA III–IV), were previously diagnosed with significant comorbidities including hypertension and diabetes mellitus and were receiving medical treatment following the guidelines of the European Society of Cardiology [18]. Nonischemic dilated cardiomyopathy was diagnosed when patients had intact coronary arteries on coronary angiography and LV systolic dysfunction (EF<40%) with a dilated non-hypertrophic LV (LVDD>55 mm) on echocardiography; furthermore, patients did not show existence of primary valvular disease.

Nine non-diseased donor hearts were used as control (CNT) samples. All donors had normal LV function and no history of myocardial disease. The hearts were considered for cardiac transplantation but were subsequently deemed unsuitable for transplantation either because of blood type or size incompatibility. The cause of death was cerebrovascular accident or motor vehicle accident.

Transmural samples were taken from near the apex of the left ventricle (maintained in 0.9% NaCl throughout the extraction procedure) and stored at 4°C for a mean time of 5.3±3.6 h from the time of coronary circulation loss.

All tissues were obtained with signed informed consent of patients. The project was approved by the local Ethics Committee (Biomedical Investigation Ethics Committee) and conducted in accordance with the guidelines of the Declaration of Helsinki.

### Homogenization of samples, electrophoresis and Western blot analysis

Fifty milligrams of frozen left ventricle was transferred into Lysing Matrix VA tubes designed for use with the FastPrep-24 homogenizer (MP Biomedicals, USA) in a total protein extraction buffer (2% SDS, 250 mM sucrose, 75 mM urea, 1 mM dithiothreitol and 50 mM Tris-HCl, pH 7.5) with protease inhibitors (25 µg/mL aprotinin and 10 µg/mL leupeptin) [19]. The homogenates were centrifuged and supernatant aliquoted. The protein content of the aliquot was determined by the Peterson's modification of the micro Lowry method using bovine serum albumin (BSA) as standard [20].

Samples were separated by Bis-Tris Midi gel electrophoresis with 4–12% polyacrylamide in a separate gel for CaM, CaN, CaMKIIδ, HDAC4, MEF2C, NFAT1 and GATA4. After electrophoresis, the proteins were transferred from the gel to a PVDF membrane by the iBlot Dry Blotting System (Invitrogen Ltd, UK) for Western blot. After blocking all night with 1% BSA in Tris buffer solution containing 0.05% Tween 20 at 4°C, membranes were incubated for 2 hours with a primary antibody in the same buffer at room temperature. The primary detection antibodies used were anti-calmodulin rabbit monoclonal antibody (1∶5000), anti-calcineurin rabbit polyclonal (1∶800), anti-NFAT1 mouse monoclonal (1∶1000), anti-HDAC4 rabbit monoclonal (1∶1000) and anti-MEF2 rabbit polyclonal (1∶800) from Abcam (Cambridge, UK), and anti-CaMKII rabbit polyclonal (1∶800) and anti-GATA4 rabbit polyclonal (1∶650) from Millipore (Lake Placid, NY, USA). Anti-β-actin monoclonal antibody (1∶1000) (Sigma-Aldrich, Missouri, USA) was used as loading control for each of the blots.

Then, the bands were visualized using an acid phosphatase-conjugated secondary antibody and nitro blue tetrazolium/5-bromo-4-chloro-3-indolyl phosphate (NBT/BCIP, Sigma) substrate system. Finally, the bands were digitalized using an image analyzer (DNR Bio-Imaging Systems) and quantified by the Gel Capture (v.4.30) and the TotalLab TL-100 (v.2008) programs.

### Fluorescence microscopy analysis

Frozen cardiac muscle sections were transferred to glass slides and fixed in cold acetone for 10 minutes at 4°C. Samples were blocked with PBS containing 1% BSA for 15 minutes at room temperature. After blocking, sections were incubated for 90 minutes at 37°C with the primary antibodies (described in Western blot analysis) in the same buffer solution, and then with FITC-conjugated secondary antibody (Santa Cruz Biotechnology Inc, Heidelberg, Germany) for 60 minutes at room temperature [19]. Sections were rinsed in PBS, mounted in Vectashield conjugated with DAPI for identifying nucleus (Vector Laboratories Ltd, UK), then were observed with an Olympus BX41 fluorescence microscope. Finally, the images were processed with ImageJ (v. 1.4.3.67) Launcher Symmetry Software.

### Electron microscopy analysis

Samples from left ventricle (size 1 mm^3^) were fixed in a solution of 1.5% glutaraldehyde plus 1% formaldehyde in 0.05 M cacodylate buffer, pH 7.4, for 60 minutes at 4°C, and postfixed in 1% OsO_4_ for 60 minutes at 4°C, dehydrated in ethanol and embedded in Epon 812. The 60 nm ultra-thin sections were mounted on nickel grids and counter-stained with 2% uranyl acetate for 20 minutes and 2.7% lead citrate for 3 minutes, for electron microscopy observation, using a Philips CM-100, with magnifications ranging from 4500 to 15000×.

### Statistical analysis

Data are presented as the mean ± standard error mean. The Kolmogorov-Smirnov test was used to analyze the distribution of the variables. Comparisons of clinical characteristics were achieved using Student's t-test for continuous variables and Fisher exact test for discrete variables. Comparisons for protein levels between two groups were performed using the Mann-Whitney U test and Spearman's correlation coefficient was performed to analyze the association between variables. Significance was assumed as p<0.05. All statistical analyzes were performed using SPSS software v. 15 for Windows (SPSS Inc., Chicago. IL, USA).

## Results

### Clinical characteristics of patients

Most of the patients were men (88%) with a mean age of 52±11 years. The clinical characteristics of patients according to aetiology of HF are summarized in Table 1. The ICM group showed a significant increase in age (p<0.01), and total cholesterol (p<0.001) compared with DCM group. Significant differences were also found in left ventricular end-systolic diameter (LVESD) (p<0.001), left ventricular end-diastolic diameter (LVEDD) (p<0.001), and left ventricular mass index (LVMI) such as an increase in the DCM group (p<0.001) compared with ICM group. The percentage of hypertensive and diabetic patients was also higher in ICM group (p<0.05 and p<0.01). Nine non-diseased donor hearts were used as CNT samples (78% male, mean age 54±8 years, and EF>50).

**Table 1:** Clinical and echocardiographic characteristics according to heart failure aetiology.

	ICM (n = 43)	DCM (n = 31)
Age (years)	56±7	48±13**
Gender male (%)	98	74**
Hemoglobin (mg/dL)	13±2	13±2
Hematocrit (%)	40±6	40±7
Total cholesterol (mg/dL)	184±48	143±42***
Serum creatinine (mg/dL)	1.2±0.8	1.1±0.5
Na (mEq/L)	136±4	135±5
NYHA class	3.4±0.4	3.3±0.5
BMI (kg/m^2^)	26±4	26±6
Prior hypertension (%)	50	27*
Prior smoking (%)	85	66
Prior diabetes mellitus (%)	48	13**
EF (%)	24±7	21±8
FS (%)	13±4	11±4*
LVESD (mm)	56±9	66±10***
LVEDD (mm)	62±9	74±12***
Left ventricle mass index (g/cm^2^)	142±36	205±63***
Duration of disease (months)	62±56	70±56

Duration of disease from diagnosis of heart failure until heart transplant.

* p<0.05;

** p<0.01;

*** p<0.001. BMI = body mass index; DCM = dilated cardiomyopathy; EF = ejection fraction; FS = fractional shortening; ICM = ischemic cardiomyopathy; LVEDD = left ventricular end diastolic diameter; LVESD = left ventricular end systolic diameter; Na = sodium; NYHA = New York Heart Association.

### Ca^2+^/CaM complex and Ca^2+^/calmodulin-dependent enzymes in heart failure

**Figure 1:**
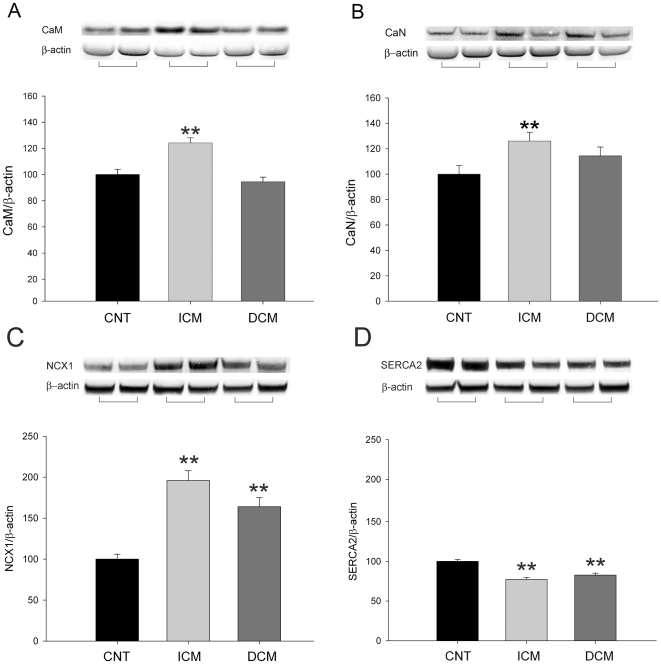
Western blots showing increased CaM, CaN and NCX1 levels, and decreased SERCA2 levels in human heart failure. CaM (A), CaN (B), NCX1 (C) and SERCA2 (D) in left ventricular myocardium from patients with ICM (n = 43) and DCM (n = 31) versus CNT group **(n = 9).** The data are expressed as means ± SEM of five independent experiments. Values are normalized to β-actin and finally to CNT group, which was also normalized to β-actin before. CaM, calmodulin; CaN, calcineurin; CNT, control; DCM, dilated cardiomyopathy; ICM, ischemic cardiomyopathy. *p<0.05 versus control.

To investigate the effect of heart failure on several key Ca^+2^ handling proteins, we determined the levels of CaM and CaN in human left ventricular myocardium by Western blot techniques. When we compared protein levels between HF (n = 74) and CNT (n = 9) hearts, the average of Ca^+2^ handling proteins (CaM and CaN) was significantly increased in pathological samples (112±3 vs. 100±6; 120±5 vs. 100±3; p<0.05 in both, when normalized to β-actin). Furthermore, Figure 1 shows that according to HF aetiology, only in ICM (n = 43) CaM and CaN were significantly increased (24%, p<0.001; and 26%, p<0.01, respectively). In addition, NCX1 and SERCA2 protein levels were also quantified. The results obtained showed that NCX1 is significantly increased in ischemic and dilated samples (96% and 64%, p<0.01, respectively) compared to controls (Figure 1C), SERCA2 showed a similar decrease in both aetiologies (23% and 17%, p<0.01, respectively) (Figure 1D).

**Figure 2:**
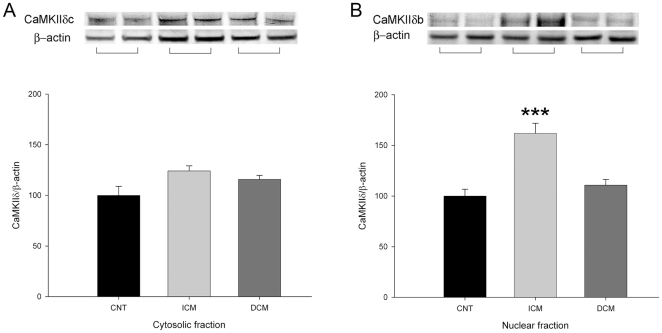
Detailed protein values of CaMKIIδ in cytosolic and nuclear fractions in human myocardium. Western blots for cytosolic (A) and nuclear (B) CaMKIIδ in controls, ischemic and dilated cardiomyopathies. The data are expressed as means ± SEM of five independent experiments. Values were normalized to β-actin and finally to control myocardium, which was also normalized to β-actin before. CaMKIIδ, Ca^2+^/calmodulin-dependent kinase II isoform delta; CNT, control, DCM, dilated cardiomyopathy; ICM, ischemic cardiomyopathy. **p<0.01 vs CNT. ***p<0.001 vs. CNT.

On the other hand, we also quantify the total CaMKII_δ_ protein amount, and its cytosolic and nuclear fractions (Figure 2). We obtained a significant increase only in ICM group for total quantity (29%, p<0.01), and for nuclear CaMKIIδb (62%, p<0.001) (Figure 2B). There were significant differences for CaM and nuclear CaMKIIδ fraction levels between these two aetiologies (p<0.01). In addition, we obtained a significant correlation between CaM protein with CaMKIIδ levels (r = 0.43, p<0.001).

### Effect of heart failure and relationship between cardiac transcriptional pathways

**Figure 3:**
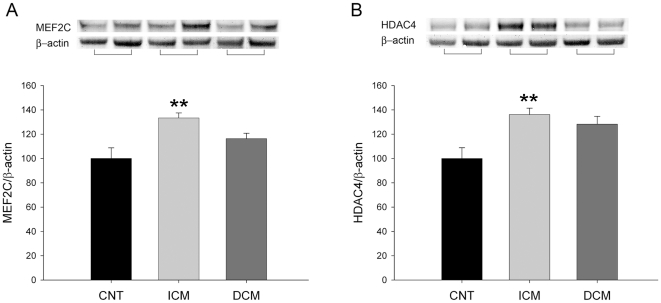
Influence of heart failure on the MEF2C and HDAC4 transcriptional factor levels. We determined the values of MEF2 and HDAC4 by Western blots. In A, values of MEF2C were significantly increased in ICM samples (n = 43). In B, similar results were obtained in LV myocardium of ICM for HDAC4. Values are expressed as mean ± SEM of five independent experiments and normalized to β-actin and finally to CNT myocardium, which was also normalized to β-actin before. ICM, ischemic cardiomyopathy; DCM, dilated cardiomyopathy; CNT, control. *p<0.05 versus CNT.

We analyzed the influence of HF on the MEF2C transcriptional factor, target of Ca^2+^/CaM signaling. We determined the values of MEF2C and HDAC4, a histone deacetylase that interacts with this factor. Pathological hearts had an increase in both proteins (126±3 vs. 100±13, p<0.05; and 133±4 vs. 100±12, p<0.05, respectively) compared to CNT samples. Then, only myocardium from hearts with ICM showed higher MEF2C and HDAC4 protein levels (33% and 36%, p<0.01, respectively) (Figure 3). When we analyzed the cytosolic and nuclear fractions of HDAC4, ICM hearts only showed a significant increase in the cytosolic fraction (45%, p<0.05) and 12% in the nuclei, but DCM did not show significant differences (16% and 24%, respectively) compared to CNT (data not shown). In addition, a statistical correlation was found between MEF2C and HDAC4 in the pathological human hearts (n = 74; r = 0.37, p<0.01). Finally, HDAC4 also showed a significant direct correlation with CaN expression (r = 0.25, p<0.05).

**Figure 4:**
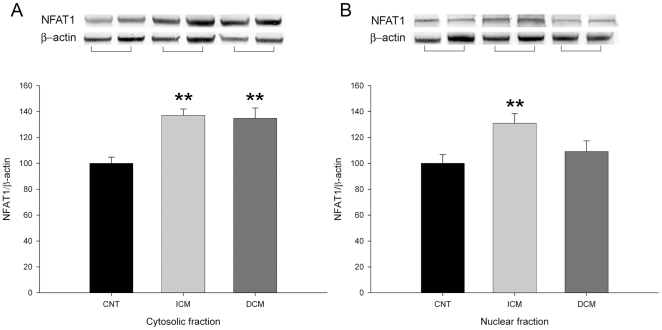
Protein levels of NFAT1 transcriptional factor in cytosolic and nuclear fractions. As shown, both distribution of NFAT1, cytoplasm (A) and nucleus (B) were increased in ICM (n = 43), but only cytosolic fraction was increased in DCM (n = 30) compared to CNT **(n = 9).** Values are expressed as mean ± SEM of five independent experiments and normalized to β-actin and finally to CNT myocardium, which was also normalized to β-actin before. ICM, ischemic cardiomyopathy; DCM, dilated cardiomyopathy; CNT, control. **p<0.01 vs CNT and ***p<0.001 vs. CNT.

Furthermore, we also analyzed whether HF induced changes in the NFAT1 transcriptional pathway. We observed a significant increase in pathological myocardium (152±7 vs 100±8, p<0.01, when normalized to β-actin). When we compared the NFAT1 according to aetiology of HF, only left ventricular myocardium from ICM hearts showed a significant increase compared to CNT hearts (66%, p<0.001) (Figure 4). Then, we quantified the protein amount of NFAT1 in cytoplasm and nucleus, and we observed that only ICM had a significant increase in nuclear NFAT1 (Figure 4B), and there were differences in nuclear NFAT1 between HF etiologies (p<0.05).

**Figure 5:**
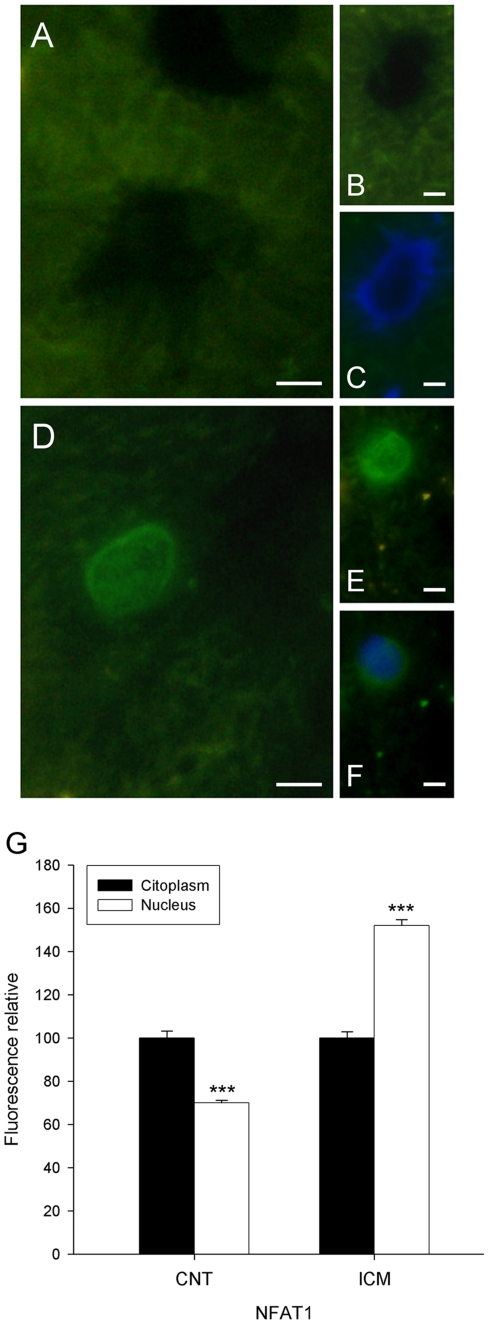
Microscopic analysis of the effect of ICM aetiology on NFAT1 nuclear translocation in human cardiomyocytes. Representative fluorescence micrographs for NFAT1 in CNT (A–C) and ICM (D–F) samples. All the micrographs correspond to four independent experiments. The bar represents 10 µm. In micrograph G, Bar graph comparing the fluorescence intensity in cytoplasm and into nucleus of NFAT1, in CNT and ICM groups. The values from the cytoplasm were set to 100. The data are expressed as mean ± SEM of five experiments. ICM, ischemic cardiomyopathy; DCM, dilated cardiomyopathy; CNT, control. ***p<0.001 versus cytoplasm.

In addition, when we analyzed the subcellular distribution of NFAT1, we can observe two distribution patterns: in the nucleus and diffused on the cytoplasm. Immunofluorescence micrographs showed that ischemic samples showed a nuclear pattern and in CNT predominates a cytoplasmatic pattern (Figure 5). Then, when we quantify the relative fluorescence of NFAT1 between cytoplasm and nucleus, ischemic samples had higher significant percentage of fluorescence of NFAT1 (52%, p<0.001) into the nucleus than outside. However, CNT samples showed a decrease in the nuclear fluorescence intensity (30%, p<0.001) (Figure 5G).

**Figure 6:**
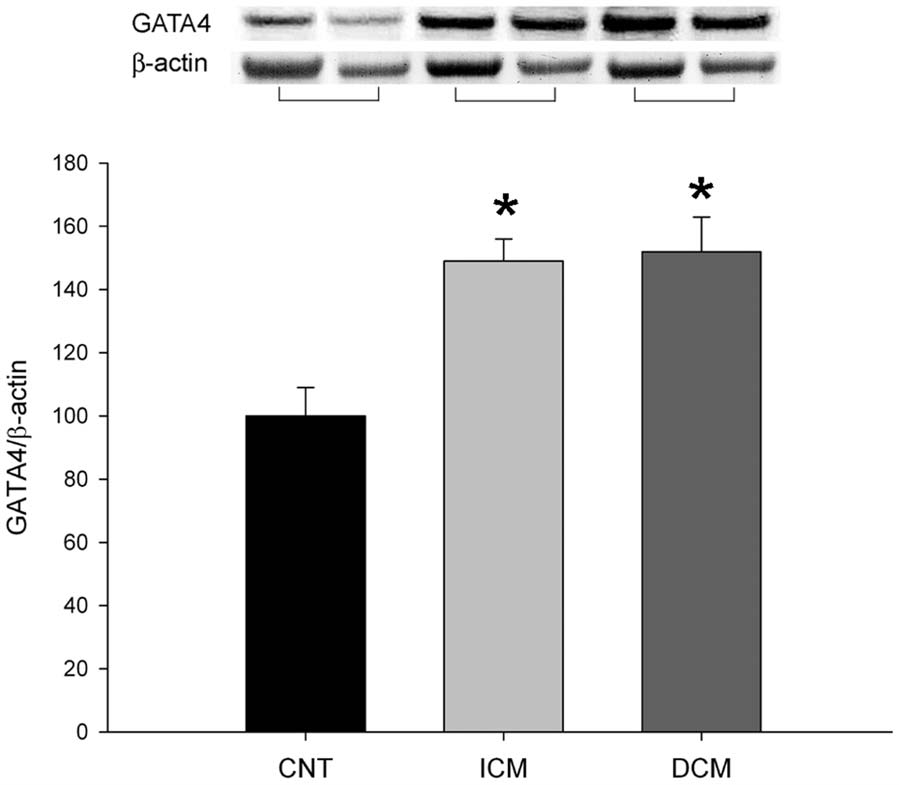
Effect of heart failure aetiology on GATA4 synthesis. As shown protein levels of GATA4 were significant increased in both aetiologies, ischemic (ICM) and dilated (DCM) cardiomyopathies compared to controls (CNT). Values are expressed as mean ± SEM of five independent experiments and normalized to β-actin and finally to CNT myocardium, which was also normalized to β-actin before. *p<0.05 versus CNT.

On the other hand, we also investigated the effect of HF on GATA4 levels in human myocardium. We found a significant increase in the levels of this factor in pathological ventricular samples (150±6 vs. 100±13, p<0.05) compared to non-failing hearts. In addition, both ICM and DCM patients showed higher GATA4 levels (49% and 52%, p<0.05, respectively) than controls (Figure 6).

**Figure 7:**
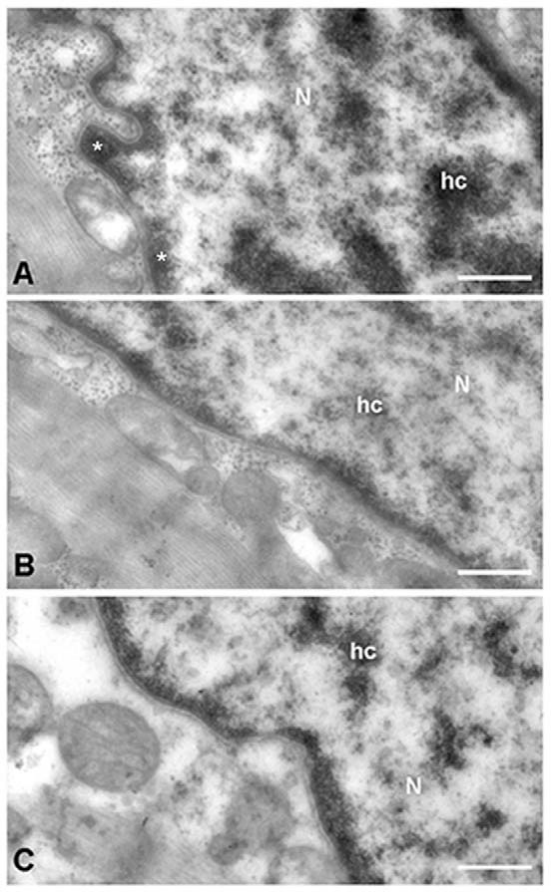
Nuclear activity as heterochromatin mass by electron microscopy in human cardiomyocytes. Cross-sectional micrographs of a nucleus in control (A), ischemic (B) and dilated (C) samples, showing a more heterochromatin condensation (hc) in controls, overall perinuclear chromatin (asterisk). N indicates nucleus. Bar represents: 400 nm.

These findings from the western blot analysis were associated with the images of human cardiomyocytes nuclei with HF, using electron microscopy (Figure 7). The masses of heterochromatin, a measure of low transcriptional activity, are more abundant in control nuclei. In ischemic cardiomyocytes (Figure 7B) there is a decrease in the percentage of perinuclear heterochromatin versus controls (Figure 7A, asterisk).

**Figure 8:**
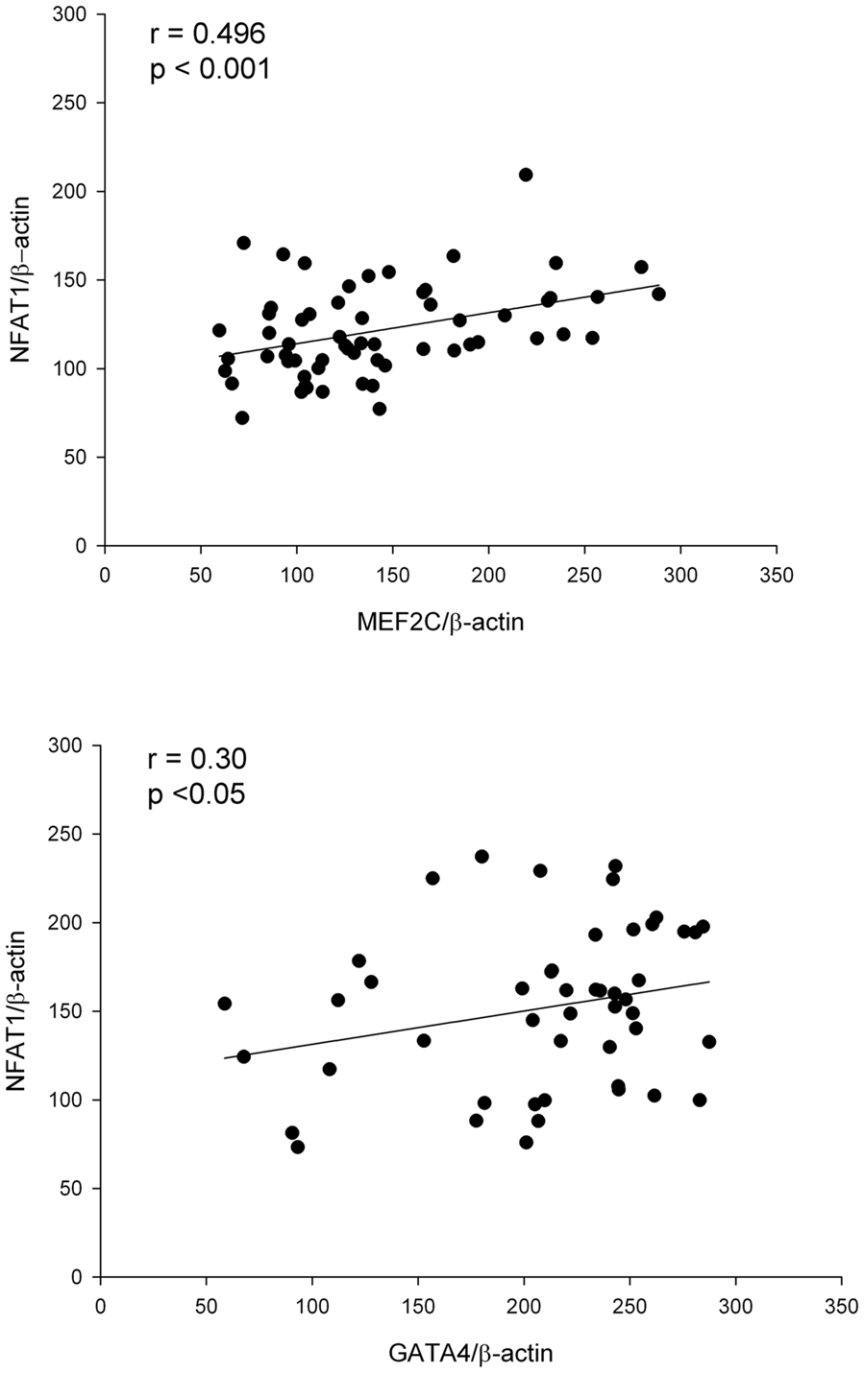
Relationship between cardiac transcriptional factors in heart failure human hearts. (A) Correlation plots between NFAT1 with MEF2 and GATA4 in pathological samples (n = 74).

Finally, we analyze the potential relationship between the different transcriptional factors for cardiac hypertrophy in the human heart. The results obtained showed that in HF samples NFAT1 protein levels were significantly correlated with MEF2 and GATA4 (p<0.001 and p<0.05, respectively) (Figure 8). Furthermore, according to HF aetiology, significant correlations between NFAT1 and MEF2 were obtained in both groups (ICM r = 0.382, p<0.05; DCM r = 0.585, p<0.01, respectively), and GATA4 protein only showed a significant correlation with NFAT1 in the ICM (r = 0.373, p<0.05).

## Discussion

This study showed a simultaneous analysis of the protein synthesis of Ca^+2^ handling machinery and the cardiac transcriptional pathways associated according to HF aetiology (ischemic or dilated) in a large group of human hearts.

Quantitative analysis of Ca^+2^ handling proteins and transcriptional factors in left ventricular samples showed an increase in pathological samples, especially in hearts from ischemic patients, and a relationship between transcriptional factor synthesis was also found. These findings would indicate that alterations in Ca^+2^ handling machinery could contribute to a phenotype of HF and support the development of many functional studies to determine which of these targets are of primary importance in this syndrome.

### Ca^+2^/CaM complex and Ca^+2^/CaM dependent enzymes in failing hearts

A number of intracellular signals are associated with an increase in intracellular Ca^2+^, consistent with a central regulatory role of Ca^2+^/CaM complex in coordinating the activities of multiple hypertrophic signaling pathways. Furthermore, Ca^+2^/CaM dependent enzymes, including CaN and CaMKIIδ, play critical and synergistic roles in the development of HF, dephosphorylating and phosphorylating several Ca^+2^-handling proteins [9], [10]. The results of the present study show a markedly increase in CaM, CaN and CaMKIIδ protein quantities in ischemic myocardium, but hearts from patients with DCM did not reach a significant increase. The difference in CaMKIIδ levels between aetiologies was due to higher elevation of this protein in the nuclear fraction in ICM than in DCM. In addition, we have also quantified SERCA2 and NCX1, two candidates that orchestrated the Ca^+2^ handling in the cardiac muscle, and in this case, we have been found them dysregulated under both pathological conditions (data not shown), as previous studies [21], [22]. Thus, it appears that in the majority of end-stage HF etiologies Ca^+2^/CaM dependent enzymes increase [23], but in our ischemic hearts the activation of these proteins is more evident than in dilated hearts, which might be important for further in vivo investigations.

### Effect of heart failure on cardiac transcriptional pathways associated with Ca^2+^ homeostasis

LV remodelling plays a critical role in the development of HF and involves LV hypertrophy and dilatation. As consequence several transcriptional factors are activated in the cardiomyocytes, among them the pathways dependent of calcium homeostasis [11]. This fact is in accordance with our results, increased levels of MEF2 and NFAT1 protein levels, and GATA4 highly expressed in cardiac myocytes [24] and regulates its target promoters in combination with these factors [9], [11], [25]. In addition, MEF2 only become active with the phosphorylation and nuclear export of HDAC4 [26] by CaN and CaMKIIδ [12]. Our data would support this fact with a significant increase in CaMKIIδ and CaN synthesis, but only in ICM hearts and mainly in the nuclear fraction, with the subsequent nuclear export of HDAC4.

On the other hand we also observed an increase in the NFAT1 protein levels in the nuclear fraction and in the distribution pattern into the nuclei by immunofluorescence only in ischemic aetiology. These findings may be in concordance with a major activation of calcineurin with the subsequent nuclear translocation of this transcriptional factor in hearts from ischemic patients. These results are supported by the electron microscopy analysis that shows a high nuclear transcriptional activity (reduction of heterochromatin masses) in ischemic hearts.

It has been argued that cardiac transcriptional pathways dependent of Ca^2+^ homeostasis in human hearts would be an important process of cardiac remodelling in HF. The present study is in line with this idea, although our results show differences between HF aetiology, a significant increase was detected only in ICM.

Perhaps, this fact could be related with the intrinsic variability of the samples, given they originate from human hearts, whose conditions (treatment they undergo) are not standardized. But it is very unlikely because in our study almost all patients received drugs like diuretics, ACE inhibitors and beta-blockers. In addition, there is precedent for specific biochemical differences between dilated and ischemic cardiomyopathies [27], [28], and it has been previously established that genes that **cause** DCM generally encode cytoskeletal and sarcomeric (contractile apparatus) proteins [29], although disturbance of calcium homeostasis also seems to be important [25].

### Association of MEF2C, NFAT1 and GATA4 cardiac transcriptional factors in human hearts

There is substantial evidence that transcriptional factors function cooperatively with each other and with coactivators and repressors in their regulation of gene expression. Specifically, *Putt et al.* provided genomic evidence for coregulation of myocardial gene expression by MEF2 and NFAT1 in advanced human HF from patients with idiopathic DCM [30]. In the present study, we determined the relationship between MEF2 and NFAT1 protein levels in the same myocardium from patients with HF, revealing a significant direct correlation in both cardiomyopathies (ischemic and dilated). These data would show that coregulation of gene expression may be also reflected at protein expression level in left ventricular myocardium. We also observed, in ICM, the correlation between the protein amounts of GATA4 with NFAT1 in ICM, a previous work where showed the interaction between both factors in transgenic mice, resulting in synergistic activation of cardiac transcription [9]. Furthermore, previous works have shown that there is cross-talk between CaMKII and CaN signaling pathways. Lu *et al.* [31] demonstrated that the transcriptional upregulation of CaN is partially mediated by CaMKIIδ in rat cardiomyocytes, and *Khoo et al.* [32] showed the role of CaMKII in CaN cardiomyopathy. Our results would be in line with the theory that there is a certain interaction between the two systems through the relationship found between HDAC4 and CaN levels, as previous reports [33].

The current study shows that the identification of an increase in the synthesis of these proteins would show that these pathways may be associated with a heart failure phenotype, especially in ischemic hearts. Furthermore, significant correlation between cardiac transcription factor protein levels, and a cross-talk between CaMKIIδ and CaN signaling pathways, HDAC4 not only may regulate MEF2 activation, would indicate the complexity of calcium homeostasis in the development HF. Therefore, considering the important role of the Ca^2+^ dependent transcritpional pathways in cardiac hypertrophy and heart failure, further studies are necessary to determine which of these targets (Ca^2+^ handling machinery and cardiac transcription factors) are of primary importance in establishing therapeutic approaches to treat patients with heart failure.
